# Public attitudes towards alcohol control policies in Scotland and England: Results from a mixed-methods study

**DOI:** 10.1016/j.socscimed.2017.01.037

**Published:** 2017-03

**Authors:** Jessica Li, Melanie Lovatt, Douglas Eadie, Fiona Dobbie, Petra Meier, John Holmes, Gerard Hastings, Anne Marie MacKintosh

**Affiliations:** a School of Health and Related Research, University of Sheffield, United Kingdom; b Faculty of Social Sciences, University of Stirling, United Kingdom; c Institute for Social Marketing, University of Stirling, United Kingdom

**Keywords:** UK, Alcohol, Telephone survey, Alcohol policy, Alcohol policy attitudes, Mixed-methods

## Abstract

The harmful effects of heavy drinking on health have been widely reported, yet public opinion on governmental responsibility for alcohol control remains divided. This study examines UK public attitudes towards alcohol policies, identifies underlying dimensions that inform these, and relationships with perceived effectiveness. A cross-sectional mixed methods study involving a telephone survey of 3477 adult drinkers aged 16–65 and sixteen focus groups with 89 adult drinkers in Scotland and England was conducted between September 2012 and February 2013. Principal components analysis (PCA) was used to reduce twelve policy statements into underlying dimensions. These dimensions were used in linear regression models examining alcohol policy support by demographics, drinking behaviour and perceptions of UK drinking and government responsibility. Findings were supplemented with a thematic analysis of focus group transcripts. A majority of survey respondents supported all alcohol policies, although the level of support varied by type of policy. Greater enforcement of laws on under-age sales and more police patrolling the streets were strongly supported while support for pricing policies and restricting access to alcohol was more divided. PCA identified four main dimensions underlying support on policies: alcohol availability, provision of health information and treatment services, alcohol pricing, and greater law enforcement. Being female, older, a moderate drinker, and holding a belief that government should do more to reduce alcohol harms were associated with higher support on all policy dimensions. Focus group data revealed findings from the survey may have presented an overly positive level of support on all policies due to differences in perceived policy effectiveness. Perceived effectiveness can help inform underlying patterns of policy support and should be considered in conjunction with standard measures of support in future research on alcohol control policies.

## Highlights

• UK alcohol policies restricting access to alcohol via pricing are the least popular.
• Females and those who are older are more supportive of restrictive alcohol policies.
• Perceived effectiveness should be considered when measuring alcohol policy support.
• Mixed methods approaches are useful to strengthen understandings of policy support.

## 1 Introduction

Alcohol use is the third leading risk factor for global disease burden (Lim et al., 2012) and accounts for an estimated £3 billion in National Health Service (NHS) costs annually within the United Kingdom (UK) (Scarborough et al., 2011). Out of concern over the scale of the economic and health burdens from alcohol-related harms (House of Commons, 2010), the UK and Scottish Governments have published strategies and implemented policies aiming to reduce harmful alcohol consumption (HM Government, 2012, Scotland and Government, 2009). Examples include minimum pricing for alcohol, greater use of brief interventions and integrating public health within the alcohol licensing system. Public support for these types of policies is varied (Banerjee et al., 2010, Wilkinson et al., 2009), but can be an important influence on political decision-making in terms of which policies are supported by governments. Negative public attitudes around a policy may lead to government withdrawing its support, as was partly the case for minimum unit pricing in England (Home Office, 2013, Lonsdale et al., 2012), and may also lead to problems with implementation and adherence (Kaskutas, 1993). Our study uses a mixed methods approach to examine public support for alcohol policy options in the UK and underlying reasons for positions taken.

Despite substantial public debate in the UK and internationally on the scale of alcohol-related harm (Plant and Plant, 2006), public opinion on governmental responsibility towards more restrictive alcohol controls and on individual policy options is divided (Tobin et al., 2011). For example, past research has shown that less intrusive lighter touch policies (e.g. education and information campaigns) or those targeting problem drinkers (e.g. treatment provision) are highly favoured while population-level alcohol policies addressing the price and availability of alcohol and directly affecting most drinkers, are less popular (Room et al., 2005). This indicates there may be latent or unobserved factors that determine support for certain types of policies. Understanding these patterns in support is particularly important since most policies that are highly supported (e.g. light-touch approaches), often have less evidence on their effectiveness compared to more restrictive policies (Babor et al., 2010, Lancaster et al., 2013).

Reasons for the incongruity of high support for ineffective policies has been given little research attention by researchers. A psychological ‘cognitive polyphasia’ explanation whereby people can hold two conflicting views, e.g. individuals can fully support specific policies while also opposing the idea of government intervening on individuals' choices (Branson et al., 2012) may offer some insight. Other studies on this topic suggest examination of moderators of policy support may lead to a partial explanation. These moderators include beliefs about whether harms are caused by alcohol, whether restrictive policies would be effective, and whom policies would affect (Kaskutas, 1993, Storvoll et al., 2014a, Storvoll et al., 2014b). Another key factor moderating policy support might be communication and understanding of the evidence base. One recent study examining support for minimum unit pricing (MUP) found that communication of potential positive outcomes of MUP may increase public acceptability (Pechey et al., 2014) and other studies have concluded that strengthening the public's beliefs in policy effectiveness would increase public support for more restrictive alcohol controls (Storvoll et al., 2014a, Storvoll et al., 2014b). In general, there have been few studies examining levels of policy support alongside examination of the moderators of this support. In particular, only on rare occasions have qualitative methods been used to understand how individuals draw on different factors when constructing views on UK alcohol policy (Banerjee et al., 2010, Cohn, 2016, Lonsdale et al., 2012). This lack of evidence was noted in a recent Drug and Alcohol Review special issue (N. Giesbrecht and Livingston, 2014) which called for further research into perceived effectiveness and public views on alcohol control in order to better understand the alcohol policy process and tackle barriers in alcohol pricing reform.

A number of conceptual approaches can be used when examining the acceptability of alcohol policies. The most common approach has been to consider support for policies as unidimensional, to be taken at face value and to be measureable using a single survey question (Branson et al., 2012). However, more theoretically-oriented approaches can be considered and three options are considered here. First, the framing of policies (how they are presented to the public) can influence how policies are understood and interpreted (e.g. social policies or health policies) and whether evidence is presented alongside them can influence public acceptability. For example, one recent study examining support for minimum unit pricing (MUP) found that communication of potential positive outcomes of MUP may increase public acceptability (Pechey et al., 2014) and other studies have concluded that strengthening the public's beliefs in policy effectiveness would increase public support for more restrictive alcohol controls (Storvoll et al., 2014a, Storvoll et al., 2014b). Second, attribution theory argues that there are inherent human biases whereby individuals may view others in poor health as responsible for their ill health because of individual choices instead of external social, structural and environmental factors (Niederdeppe et al., 2008). Thus attribution theory suggests individuals may be more willing to support policies targeted at those they perceive to have drinking problems and oppose interventions that directly affect their own lives. Third, the interactionist approach argues that it is through interactions with other people that a view on policies is developed and confirmed (Cohn, 2016). Adopting an interactionist approach would allow policy support to be examined as positions that are shaped by a dynamic process rather than a static attitude. A recent study that adopted this approach found that public acceptability towards alcohol policy was not a singular view based on an economic rationalisation of costs and benefits of each policy, but was instead a dynamic process that emerged through exchanging views with others and contextualising policies within specific social settings (Cohn, 2016). It is in this context that our study aims to apply a concurrent mixed methods approach to 1) examine the underlying structure of alcohol control policy support in relation to demographics, drinking behaviour and public perceptions of UK drinking and government responsibility over alcohol related harms, and 2) explore how perceived effectiveness can influence and/or inform quantitative understandings of these dimensions of policy support.

## 2 Methods

### 2.1 Data

Data used in this study came from the Alcohol Policy Interventions in Scotland and England project (APISE) and consisted of a cross-sectional telephone survey and focus groups. APISE is the UK arm of the International Alcohol Control Study, an international collaboration examining the effectiveness of alcohol control policies via survey data and cross-country comparative analyses (Casswell et al., 2012). Ethical approval for the telephone survey and focus groups was granted by the Universities of Stirling and Sheffield.

### 2.2 Quantitative APISE survey

The first wave of APISE was conducted by an independent market research company and surveyed 3477 drinkers in Scotland (n = 1728) and England (n = 1749), using Computer Assisted Telephone Interviewing (CATI) between September 2012 and February 2013. Landline telephone numbers were selected through list-assisted random digit dialling. Upon contact with a household, the number of eligible adults (aged 16–65) in the household was determined. As the UK minimum legal purchase age for alcohol is 18, the sample included drinkers who were not able to purchase alcohol legally. In households with more than one eligible adult the respondent was randomly selected using an adapted Rizzo method (Rizzo et al., 2004). Final eligibility was determined if the selected respondent had drunk any alcohol in the last six months. Based on American Association for Public Opinion Research (AAPOR) recommendations (The American Association for Public Opinion Research, 2011), the response rates (RR3) were 16% (England) and 19% (Scotland).

### 2.3 Survey measures

Survey respondents were asked questions (validated through cognitive interviewing and testing) regarding their demographic characteristics, perceptions around UK drinking and government responsibility over alcohol related harms, and alcohol consumption (Table 1). Other moderators of policy support (e.g. perceived effectiveness and whom policies would affect) were measured within the qualitative component of the study. Respondents' perceptions towards drinking in their country were assessed through their answers to the following questions (based on a five point scale): ‘Thinking about everything that happens around you, would you say that people in Scotland/England are generally discouraged from drinking alcohol or encouraged to drink alcohol?’; ‘Would you say that people in Scotland/England have a very unhealthy relationship with alcohol or a very healthy relationship with alcohol?’. Views on government responsibility were measured via their level of agreement with the following statement: ‘The government should do more to tackle the harm done by alcohol’. Alcohol consumption was measured through a beverage and location specific quantity-frequency measure which first asked respondents how often they drank alcohol at fourteen separate locations (e.g. own home, pubs, restaurants, etc.), then for each location reported, the types of alcohol they would consume on a typical occasion and how much of each type they would consume.

Support for alcohol policies was measured through twelve alcohol policy statements (Table 3) rated on a 5 point Likert scale (strongly support to strongly oppose). To maximise the policies measured the sample was randomly divided by the CATI programme into two groups (Group A and Group B) with equal representation of Scottish and English drinkers. Each group rated seven different alcohol policy statements. Two statements on price policy (increasing prices and pricing based on strength) were included in both groups as minimum unit pricing was a high profile policy area for both the UK and Scottish governments at the time of questionnaire development (HM Government, 2012, Scotland and Government, 2009).

### 2.4 Statistical analyses

Weighting was used to adjust for the unequal probability of selection of individuals into the sample and to reflect the age, gender, and working status of English and Scottish drinkers separately. English and Scottish data were merged for analysis and further post-weighting by country was applied to reflect differences in drinker population sizes. Respondents who reported consuming alcohol in the past 6 months but had inadequate or missing data on alcohol consumption measures (n = 17) were excluded from the analyses, leaving a total sample size of 3460.

Drinking categories were created based on thresholds commonly used in the UK (National Institute for Health and Care Excellence, 2010); Moderate drinkers were defined as men consuming ≤ 21 units/week and women consuming ≤ 14 units/week (1 UK alcohol unit = 8g/10 ml ethanol), hazardous drinkers as men consuming > 21 and ≤ 50 units/week and women > 14 and ≤ 35 units/week, and harmful drinkers as men consuming > 50 units/week and women consuming > 35 units/week. Annual household income before taxes was collapsed into three categories: low (<£20,800), middle (£20,800–41,599), and high (£41,600+). Information on occupation was collected based on the 2010 National Statistics Socio-economic Classification (NS-SEC). Due to a high proportion of missing responses on income and occupation (22–24% of the sample), missing data were included as separate categories in multivariate analyses. Chi-square analyses indicate missing data on income and occupation was more common among females and younger people (p < 0.05).

Principal components analysis (PCA) is a statistical technique that utilizes correlations between a set of variables to identify unobserved factors (i.e. principal components or dimensions) which drive patterns of response to those variables (Agresti and Finlay, 2009). In this case we sought to identify dimensions characterising the types of policies that received similar support. Using SPSS 20.0 for Windows, PCA using varimax rotation was conducted to transform each set of seven alcohol policies within Groups A and B into new principal components (using an eigenvalue of 1 as a cut-off). Applying a method which has been used elsewhere (Callinan et al., 2013, Wilkinson et al., 2009), average support for each policy item within each component was then summed and divided by the number of policies within each component so that a score of 1 indicated that all policy items within that dimension were strongly opposed while a score of 5 indicated all policies were strongly supported. These new mean scores for each respondent were used as dependent variables in multiple linear regression models to analyse how demographics, perceptions, and drinking behaviours were associated with each dimension of policy support. Categorical predictors for regression models were recoded and entered as dummy variables (low vs. high income, missing income, education, occupation, and missing occupation, no vs. yes children, moderate/hazardous vs. harmful drinkers).

### 2.5 Qualitative focus groups

Concurrent with the survey, sixteen focus groups with adult drinkers (aged 16–65 and drank within the last six months) from the general population (separate from survey sample) in England (n = 45) and Scotland (n = 44) were conducted between October and November 2012. These explored drinkers' awareness of, response to, and support for a range of alcohol control policies. A quota sample based on age, gender and socio-economic background (measured using occupation of the household's highest earner) was recruited by independent market research recruiters who either approached individuals on the street or knocked on people's doors within the sampling areas. Recruiters used a structured screening questionnaire to assess the eligibility of potential participants (Table 2). Individuals who were interested in taking part were provided with an information sheet and consent form, which was then returned before the focus groups took place. Focus groups consisting of four to six participants were conducted by ML, JL (England) and DE (Scotland) and took place in a variety of neutral settings (e.g. community halls, hotels) across four locations in England and four locations in Scotland. Participants received £25 for taking part in the study.

We developed a two-part semi-structured topic guide with open-ended questions exploring key policies and related themes identified within the literature. In the first part, general questions were used to explore participants' attitudes towards alcohol and their opinions on the problems caused by alcohol in their country; in the second part, thirty-three alcohol policies were presented to participants through a series of statement cards, each with a different alcohol policy (Appendix A). To allow for detailed discussion, a selection of policies (i.e. not all thirty-three policy statements) was presented to different focus groups to ensure that various types of policies from all thirty-three statements were covered across all groups (Appendix A). In addition to effectiveness, policymakers are interested in the potential appeal of policies as knowing the types of policies the public likes can inform how they frame and implement certain policies. We therefore asked participants to comment and place each policy on a matrix (Appendix B) which indicated the extent to which they liked/disliked the policy and how effective/ineffective they thought it would be. If there was disagreement or uncertainty on where to place policies, facilitators asked additional prompting questions and then either asked for a final vote or suggested a place for the policy on the matrix that was half way between liking and disliking or effective and ineffective, checking there was consensus before placing each statement on the matrix. Focus groups were audio recorded and transcribed verbatim (due to a technical failure with the audio recording equipment focus group E8 relied on researcher field notes for the analysis).

### 2.6 Focus group analysis

Fourteen of the thirty-three policies examined in the focus groups were comparable to the survey and explored in this paper. Using an inductive approach, data were initially coded according to emerging themes that related to policy topics and statements used to guide discussions within focus groups and the nature of alcohol problems and associated factors. Through an iterative process, a coding frame was developed and refined as more data was analysed. Using Nvivo V10, DE identified themes through close reading of the transcripts and confirmed with ML for accuracy and consistency. DE, FD, LM and ML then created summaries for each of the reasons for support or opposition to alcohol control policy statements, which identified the varying attitudes expressed by participants towards the policies and participants' perceptions of the policies' effectiveness. Relevant findings were selected and presented in reference to the survey findings for this study.

## 3 Results

### 3.1 Quantitative findings

Sample characteristics for Groups A and B are presented in Table 1. The average age of the overall sample was 40 years (SD = 13.90) and a small majority (59%) were classed as moderate drinkers, although a higher proportion of hazardous and harmful drinkers were identified than in other UK surveys, probably due to the more detailed survey measure used (Casswell et al., 2012). Although the majority believed that government should do more to tackle alcohol harms (72%), they were slightly more divided on whether people were encouraged to drink (56%) and had an unhealthy relationship with alcohol (59%).

Mean scores for each of the policy statements in Groups A and B are shown in Table 3 with a higher score indicating a stronger level of support. Overall, both groups were in support of almost all of the alcohol policies, although there is substantial variation in the level of support. Greater enforcement of under-age sales laws, more police patrolling the streets, doctors screening patients, and public health campaigns for hazardous drinking were strongly supported (mean > 4.0). Support was more divided on regulatory policies such as earlier closing times, restricting the number of outlets, reducing the drink driving limit and pricing based on strength (mean between 3.0 and 3.6). The least favoured policy was increasing the price of alcohol (mean < 3.0).

PCA indicates that there were two underlying dimensions for policy support in Groups A and B, although the different policies in each group, and potentially the different samples, meant the dimensions are also slightly different across groups (Table 4). The first dimension in Group A related to controls on economic, spatial and temporal aspects of *alcohol availability* and included four policies where responses were correlated with one another: restrictions on outlets selling alcohol, earlier closing times, increasing the price of alcohol and alcohol pricing based on strength. The second dimension focused *on provision of health information and treatment services* and included three policies: warning labels on alcohol products, public information campaigns, and more treatment services. In Group B, the first dimension specifically related to alcohol *pricing* and included two policies on increasing price and pricing based on strength. The second dimension could potentially include a wide range of policies, half of them however had low factor loadings (<0.5) (Table 4). Adopting a stricter criteria of only accepting eigenvalues greater than 0.7 (Jolliffe, 1973) produced four dimensions separate to the pricing dimension, three of which contained only one policy: reducing drink driving; ban of drinking on public transport; and doctors' screening. Based on this, these policies were excluded in a new PCA containing only those policies with high factor loadings. This produced a more reliable second dimension (alpha score = 0.609) which related to *greater law enforcement*, comprising two policies - more police patrolling streets and greater enforcement of laws on underage sales (factor loadings 0.853 and 0.833 respectively). Results corresponding to this more strictly defined dimension are presented in the remainder of this paper. Mean support scores for each dimension show that support was strongest for policies increasing law enforcement and providing health information/treatment services (4.32 and 4.04 respectively) and more divided for pricing and availability pricing policies (3.12 and 3.12).

Regression models were fitted for each policy dimension (Table 5). After controlling for all factors, standardised betas indicate that belief that government should do more to tackle alcohol-related harm was the strongest predictor of support across all policy dimensions. Being female, older, and drinking at moderate levels were also positively associated with higher support for all policy dimensions. Being female was a particularly strong predictor for more support on availability policies, while older age was a strong predictor on support for greater enforcement of laws. Belief that people were encouraged to drink alcohol and that Scotland/England had an unhealthy relationship with alcohol was a strong predictor for higher support on policies restricting availability and provision for more health information and treatment services. For socioeconomic status, associations varied depending on which measure was used. For education, those with higher level education were less likely to support policies reducing the availability of alcohol and greater enforcement of laws, but more likely to support pricing policies. Whereas those with higher income were less likely to support policies reducing availability and those related to providing health information and treatment services. Compared to Scottish drinkers, English drinkers were more likely to be supportive of policies restricting availability.

### 3.2 Qualitative findings

Data from the focus groups help validate and explain findings from the quantitative analyses by providing further insight into two underlying influences on policy support: how much the policy was liked and its perceived effectiveness. Using the photographs we took of the policy matrices as an analytical tool to guide our initial comparison of the survey and focus group data, we engaged in more in-depth analysis by using transcript data to understand how and why responses aligned or differed. Policies are presented in similar groupings to the dimensions identified in the quantitative analyses: availability (pricing and number of outlets); provision of health information and treatment services (health information campaigns, labelling, and treatment services); reducing anti-social behaviours and others (greater law enforcement, penalties for underage sales, doctors screening, etc.)

#### 3.2.1 Restricting availability

Similarly to the survey, focus group participants were generally less favourable towards policies restricting the availability of alcohol compared to the other policies. This was because of their perceived impact on everyone in the population and a general sense that other problematic drinkers would circumvent or ignore availability restrictions.

Pricing policies were commonly disliked. While some participants identified cheap alcohol prices as a contributing factor to problems caused by alcohol, many disliked the idea of increasing prices through taxes or MUP because they considered it was unfair to the average responsible drinker: ‘There again that's not fair on us lot that is probably sensible. Why should we pay more…?’ (E5). Some participants also believed increasing prices could lead to unintended consequences such as increased crime through people stealing alcohol and bootlegging, or increased use of alternative drugs.

MUP was also broadly disliked as there was scepticism over motivations for increasing the price and how the additional money from alcohol sales would be used:

… money that is generated from this minimum pricing doesn't go to the Government it just goes to Asda (supermarket) which I think is ridiculous … actually put it into sort of educating people and sorting these folk out. (S6)

Awareness of MUP was considerably higher in the Scottish groups given its passing earlier that year. Despite its widespread dislike, many in both groups thought that MUP could potentially be effective in reducing other people's alcohol consumption: ‘I know for a fact it is a dislike but I think it is effective’ (E2). This was because there was an acknowledgement that cheap alcohol prices could influence drinking behaviours: ‘I think it would be effective because then people won't be so inclined to go to the stronger spirits and get as hammered you know if they are going to spend a lot of money’ (S5).

Allowing local licensing authorities to block license applications based on public health concerns generated uncertainty over its effect on drinking behaviours. Some felt it would have little effect because there were already many licensed outlets and ‘regardless of how many shops there are you are still going to buy alcohol’ (E5). Earlier closing times in off-licenses/supermarkets were thought to be too much of an inconvenience for some participants and would be ineffective in reducing drinking as people would ‘just get it earlier in the day’ (S1) or ‘buy more there and then’ (E2) to stock up before stores closed.

#### 3.2.2 Provision of health information and treatment services

Overall, support for policies aimed at providing health information and treatment services was more varied than in the survey. While the survey found high support for policies related to public health campaigns, alcohol labelling and treatment services, focus group data suggests this may have been driven more by a liking for the policy than its perceived effectiveness. For example, participants were generally positively disposed towards public health information campaigns, however they questioned their effectiveness: ‘there is hundreds of that about and nobody really cares, nobody pays attention’ (S4). One participant felt that personal experience instead had a potentially larger influence on drinking behaviour: ‘I think you would have to see it first hand and experience the effects of it rather than somebody putting a poster in front of you … ’(S3).

In contrast to the quantitative findings, alcohol warning labels were mostly unsupported. This was perhaps because participants assumed labelling would be similar to the labelling used on cigarettes which was thought to have been ineffective in changing behaviour: ‘I don't think it shocks people anymore when they see it because they get so used to it’ (E1). Some also thought alcohol warning labels would be excessive because they did not view drinking to be as harmful as smoking: ‘Smoking is bad for you full stop whereas if you are drinking but not to excess it's not as extreme … it's kind of over the top to put it on the packaging’ (S6).

Participants were more divided on increased funding for treatment services for dependent drinkers. Some felt it was a good idea, ‘I think if someone has got a problem then help’ (E4), but there was also a perception that services were often a waste of public resources and money, with those receiving treatment frequently relapsing. Underlying some of these comments was a feeling that it was the individual who was to blame for their problem; ‘I know people say that it’s an illness, but they get themselves into that state in the first place’ (S3), as well as concern over where the additional funding would come from. This lack of consensus contrasts with the more favourable support within the survey and indicates that though some may believe treatment in principle is beneficial, others may be unconvinced of its overall effectiveness and less sympathetic towards dependent drinkers.

#### 3.2.3 Reducing anti-social behaviours (ASB) and other policies

Social harms to others, particularly violence accompanying binge drinking, were commonly reported as significant problems caused by alcohol across all focus groups. Because of this, there was higher appeal for policies aiming to reduce anti-social behaviours (ASB). For example, participants supported more policing on streets when bars/pubs closed: “I definitely do think there should be more on a night out … when everyone is waiting for the taxis that's when it all starts” (E3). However, there were concerns over barriers in implementation such as limited resources ‘ … they are cutting them all back, how are they going to get more patrol on the streets when they've not got any on patrol?’ (S8) and poor satisfaction with police competency ‘police these days they are useless they don't do nothing’ (E3).

Participants felt that most existing retailer schemes to discourage under age sales (Appendix A), particularly those in supermarkets (e.g. Challenge 25), were already effective and introducing new ones or having tougher penalties would be largely ineffective as young people could acquire alcohol through alternative sources (e.g. family, friends). Support for banning drinking on public transportation was divided based on participants' attitudes towards drinking on trains. Some felt it would be unfair on professionals commuting from work who drank responsibly while others viewed drinking on trains as entirely unnecessary. Overall, there was a wider concern that ‘drinking on public transport isn't the problem, it's drunk people being abusive and threatening on public transport that is the problem’ (E6). Because of this, banning only drunk people from travelling on public transport was more supported than a complete ban on drinking.

Lastly, support for doctors screening patients was mixed. Some liked the policy in theory because they felt that because of doctors' authority, patients would be receptive to receiving information from them and reflecting on their own alcohol consumption. However practical issues were identified, such as whether doctors would have time to ask about drinking, and if they did, whether patients would actually act on any advice or potentially lie to doctors about their own drinking: ‘whatever they say to the doctor you could probably double it’ (S7).

#### 3.2.4 Othering

A dominant theme to emerge was that participants tended to assess support for policies by focusing on the impact on problematic ‘others,’ particularly harmful drinkers and youthful drinkers, while simultaneously rejecting policies that might impact on their own drinking. This inclination to identify others as different to one's self is known as othering (Johnson et al., 2004) and is a concept that has been applied elsewhere to understand how people might predict others' responses to different health interventions (Thompson & Kumar, 2011). Othering in this context led to greater support for policies that targeted ‘problem’ drinkers and was particularly evident among pricing policies. Targeting problem drinkers was often perceived as futile because of the belief that they would always find ways to acquire alcohol. Othering was also evident in terms of age. When prompted to think about which groups experienced the most drinking problems, many participants, including younger ones, identified young people: ‘I just tend to find that more young ones are drinking these days and that's a problem, you go out in the street and there is just crowds of them’ (E1). This perception was further reflected in participants' interpretation of how certain policies might be more effective on younger age groups:

I know that for a lot of people it doesn't make any difference, but if you think of the sort of people who might be drunk … I mean I am just thinking of younger folk they'd be thinking let's go and get some big cheap bottles of cider (S7) (Earlier closing times)

For an eighteen year old kid you would have to have ‘drinking alcohol makes you crap with the ladies’ … that's the only warning they are going to listen to (S5) (Warning labels on alcohol products)

I can see that being good for maybe, like effective in terms of young folk drinking because it affects all the stuff that they drink (S4) (MUP)

This focus on younger drinkers in particular may help explain some of the positive age effects identified in the quantitative analysis.

## 4 Discussion

Our study is the first mixed methods analysis of underlying dimensions of UK alcohol policy support and the relationship between these and perceived policy effectiveness. We identified four underlying dimensions that shaped support for different types of alcohol control interventions. Within the survey, policies aimed at reducing harms and enforcing laws were strongly supported, while pricing policies and those restricting availability were less favoured, a common finding elsewhere (Diepeveen et al., 2013, Nelson et al., 2015, Room et al., 2005). Those who were most supportive of alcohol control policies were females, older people and those who drank less, which is consistent with many findings from Australia, North America, and western European countries (Giesbrecht et al., 2007, Holmila et al., 2009, Latimer et al., 2001, Wallin and Andreasson, 2005, Wilkinson et al., 2009). While some studies have found associations between socio-economic status and policy support, we found that associations varied depending on the socioeconomic measure used and identified stronger links for attitudes around drinking and government responsibility.

Research on policy attitudes has typically used opinion poll measures (e.g. binary support vs. oppose responses or Likert scales) to capture the level of support for policies. Our study highlights the limitations of using only these unidimensional measures for understanding support for control policies and how sensitive levels of support can depend on the data collection method used. There were nuanced differences in support between particular policies for which we were able to develop greater understanding through more in depth discussion of policies and perceived effectiveness within focus groups. We anticipated that insights from framing perspectives, attribution theory and interactionist approaches would help shed light on underlying factors affecting public opinion.

With regard to framing, the UK Government described policies in its UK Alcohol Strategy in relation to its need to address the issue of binge drinking even though policies presented in the strategy were wide-ranging and tackled many different issues (e.g. health, economic, and social problems) (HM Government, 2012). According to the Government, ‘binge drinking’ was the critical problem that Britain needed to address. This type of framing can potentially affect individuals' interpretations of each respective policy as they may define binge drinking differently or associate it with different types of issues and subsequently rank policies based on these perceptions. Furthermore, views on drinking practices and culture that frame perceptions of ‘responsible’ and irresponsible drinking could inform attitudes towards policies. Within our study, survey respondents were prompted with the statement, *“Now we have some questions about whether you support or oppose different ways that alcohol could be dealt with in society. How strongly would you support or oppose the following …”* while focus groups were told the following statement, *“I'd now like to discuss some of the ways government, local authorities and regulators have tried or have thought about trying to reduce alcohol consumption.”* The survey prompt was more general and by its categorical nature, primed respondents to think of policies in mainly binary ways (support or oppose) whereas the second prompt used in the focus groups was more open ended and framed policies in relation to governmental controls and ways to reduce alcohol consumption. This difference in framing, in addition to discussion around perceived effectiveness, could help explain some of the discrepancies in support (e.g. MUP) found between the quantitative and qualitative analyses. But more importantly it illustrates the influence of who is presenting the policy (researchers, government) on interpretation and how policies can be framed differently to audiences.

A more central theoretical approach that emerges from analyses is attribution theory in relation to the othering theme identified in the focus groups. Attribution theory is a useful tool in understanding how participants were more likely to support policies that would have less of a direct effect on their own drinking participants (e.g. more treatment services). Our qualitative data show that attitudes toward control policies are affected by individuals' own identity (e.g. if they saw themselves as responsible drinkers) and how they view others around them. This was most apparent when participants justified their disapproval for pricing policies because they believed these types of policies would unfairly punish responsible drinkers. Heavy drinkers or ‘others’ were often viewed as to blame for their own drinking problems without consideration of other structural factors (e.g. number of alcohol outlets, closing times). Policy support is shaped by individuals' attitudes and the social context in which attitudes towards others are formed. Our findings revealed that there were perceptions that many alcohol problems result from the behaviours of a minority of the population. Consequently population-level restrictions on alcohol availability, which have been shown to be the most effective in reducing alcohol consumption (Babor et al., 2010), may be less popular policies compared to others targeting certain groups (e.g. under age,alcoholics) independent of perceived effectiveness or liking of the policy because of this othering and attribution effect.

Finally, adopting an interactionist perspective to our qualitative data uncovers that policy support is not merely a unidimensional measure of support or a rational decision based on perceived effectiveness but is indeed multifaceted and affected through the interaction with others. It was subsequent discussions around moderators of support that shed light onto a number of factors affecting policy attitudes such as the level of personal intrusion (e.g. pricing), which groups are perceived to be problematic (othering), unintended consequences (e.g. increased crime), barriers to implementation (e.g. not enough funding) and experiences of current and past policies (e.g. closing hours, labelling). These discussions influenced how policies were interpreted and supported within the group. For respondents, determining acceptability was therefore a process rather than an event leading to a simple and static supportive/unsupportive position (Branson et al., 2012, Cohn, 2016). Discussions also helped participants reflect on the social context within which these policies would be implemented. For instance, the feasibility of implementing certain policies was thought to depend on the costs of resources needed e.g. more law enforcement patrolling streets for ASB within communities or treatment services. Perceptions may also be shaped by whether policies have already been introduced. Participants often rationalised support for labelling or increasing taxes in comparison to laws on cigarette smoking. Past research has shown that opposition towards a policy can decline once a policy has been introduced (Diepeveen et al., 2013) as was the case with the ban on smoking in public spaces within the UK and Ireland (Branson et al., 2012).

Focus group data revealed that support for alcohol policies is complex. Though there were some demographic themes that indirectly supported survey findings (e.g. older age groups being more supportive of specific policies because they believed younger age groups were the problem), a more substantial finding was that perceiving a policy to be effective does not necessarily lead to liking the policy (e.g. MUP), and vice versa (e.g. health information). This disparity in level of appeal and evidence challenges the notion that individuals make rational decisions based on evidence. The ‘intervention ladder’ proposed by the Nuffield Council of Bioethics is a device for comparing policy options based on their level of intrusion on individuals' liberties (“Public Health: Ethical Issues”, 2007); the more intrusive the policy, the stronger the justification has to be. Within this thinking is the belief that if there is a clear indication that a restrictive policy will produce the desired effect, people will be willing to accept it and be favourable towards the policy regardless of any loss of liberty. Findings from our focus groups however challenge this way of thinking as participants would at times express opposition towards some policies despite perceived effectiveness (e.g. MUP). Therefore measuring support and perceived effectiveness alone may not be sufficient to understanding overall attitudes towards policies since one does not necessarily predict the other. Instead, support for a policy is more fluid and affected through competing views and interactions with others. It was through group discussion around various moderators of support (where the problems lie, who is affected, how are drinking cultures cultivated, who should take responsibility etc.) that participants' positions for policies were conceived, shaped, and confirmed.

There are some important limitations to consider. Firstly, both our quantitative and qualitative samples consist of only drinkers. Therefore, any non-drinkers, ex-drinkers or occasional drinkers who have not drunk in the last six months were excluded from our analyses. Previous studies have identified a strong negative relationship between policy support and alcohol consumption (Macdonald et al., 2011), suggesting our findings are more likely to underestimate policy support. Our telephone survey also only recruited through landlines, excluding those with only mobile phones. This could potentially misrepresent the drinker population. However recent research has found this issue is less problematic once demographic weighting is undertaken (Livingston et al., 2013), which has been done in our study. The response rates were also low (though this is more common in telephone based surveys (Kempf and Remington, 2007) and our rates are comparable to other similar surveys (Livingston et al., 2013)), and we cannot assess the potential impact of this on non-response bias. This may impact the generalisability of our findings to the wider drinker population and findings should be interpreted with caution as a result. However we have randomly sampled and weighted our data based on the drinker profiles from national surveys (Health Survey for England and Scottish Health Survey) to ensure as representative a sample as possible. Other work has also suggested that there is little evidence of bias in estimates of policy support when adjusting for non-responders (Maclennan et al., 2012). Moreover, the study has considerable strengths as the first mixed methods research on this topic within the UK. The within-sample analyses suggest latent structures underpin support for alcohol policies and the focus group research begins to unpack this patterning of support and indicates important avenues for future research on attitudes to public health policy. A final limitation is that some policies presented in the survey were not directly comparable with focus group policies (e.g. greater enforcement of underage sales), though these differences were minor.

Our findings reflect the complexities of understanding public opinion of alcohol control policies. Policy support is not unidimensional, nor is it solely determined by one factor such as perceived effectiveness. Instead there are dialectical relationships between social (e.g. policy stakeholders, the media, researchers, the public) and cultural determinants (e.g. drinking culture and practices, othering) that converge with policy support and should be examined in further studies. While qualitative findings supported the dimensions and general patterns identified within quantitative analyses, the degree to which policies were supported within the survey and focus groups differed. Therefore the high policy support found within the survey should not be over-interpreted given that perceptions of the effectiveness of most alcohol control policies found in our focus groups were fairly mixed and did not necessarily always align with policy support. Future comparable surveys should consider the potential for acquiescence bias (Bowling, 2005) and interpret estimates with caution. When measuring alcohol policy support, future research should also consider multiple factors of support (e.g. perceived effectiveness, appeal, othering, etc.) rather than a simple construct of support versus oppose as this will lead to a fuller understanding of public opinion towards alcohol control interventions and why particular policies may lack political acceptability.

## 5 Conclusion

Overall we found that drinkers are more supportive of less intrusive alcohol policies aimed at reducing alcohol-related social and health harms than policies restricting their own access to alcohol and these dimensions of support are patterned by gender, age, and drinking behaviours. Analyses of alcohol policy support should also account for perceived effectiveness of interventions as this is an important factor that may inform support but is not consistently aligned with how much the public like a policy. Further mixed-methods as well as longitudinal studies are needed to help map out relationships between drinking behaviour, alcohol attitudes, perceptions, and policy support.

## Figures and Tables

**Table:** 

Focus Group^a^	Alcohol policy statements
E1, E3, E5, E7,S2, S6, S7, S8	Doctors or health professionals ask patients about their drinking habits and, where necessary, offer advice on how to reduce their alcohol consumption
E2, E4, E8, S1, S3, S4, S5	Fund more treatment services for dependent drinkers
E4, E6, E8, S1, S3, S4, S5	Public information campaigns warning of the harms to health that can be caused by alcohol
E1, E3, E5, E7, S1, S2, S6, S7, S8	Labels on alcohol products warning of the harms caused by alcohol
E1, E2, E3, E4, E5, E6, E7, E8, S1, S2, S3, S4, S5, S6, S7, S8	Introduce minimum unit pricing - Price drinks based on alcohol strength so that the more alcohol a drink contains, the more it costs (*e.g. minimum of 50p per unit*)
E1, E3, E5, E7, E8, S1, S2, S6, S7, S8	Increase alcohol taxes by 2% more than inflation each year
E1, E3, E5, E7, S1, S2, S6, S7, S8	Introduce more retailer schemes to check the age of people buying alcohol (*e.g. ‘Challenge 25’*)
E2, E4, E6, E8, S1, S3, S4, S5	Tougher penalties on shops who sell alcohol to people under-age
E3, E5, E6, E7, S2, S6, S7, S8	Allow local licensing authorities to block license applications if they believe having more alcohol outlets in the area will damage the public's health
E1, E2, E4, E8, S1, S3, S4, S5	Earlier closing times for buying alcohol from off-licenses and supermarkets
E1, E3, E5, E7, S1, S2, S6, S7, S8	More police patrolling streets when bars and night clubs close
E1, E3, E5, E7, S2, S3, S6, S7, S8	Ban people who are drunk from travelling on public transport
E1, E2, E4, E8, S1, S3, S4, S5,S8	Banning drinking on trains after 9pm
E2, E4, E6, S1, S3, S4, S5, S8	A complete ban on drinking on public transport

a See Table 1 for further details on focus group numbers; ‘E’ denotes English groups, ‘S’ denotes Scottish groups. While focus groups examined 33 statements, only fourteen of the policies comparable to the APISE survey were analysed in this paper and presented here.

**Figure:**
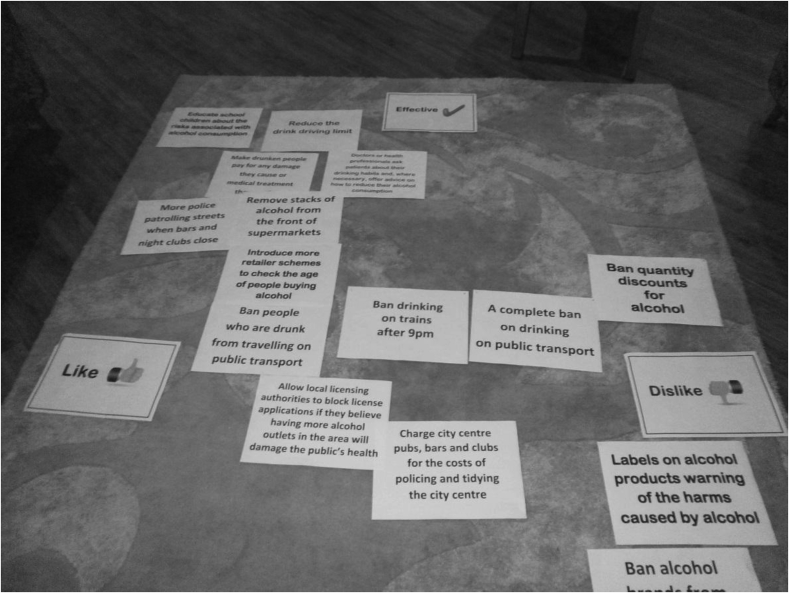


**Figure:**
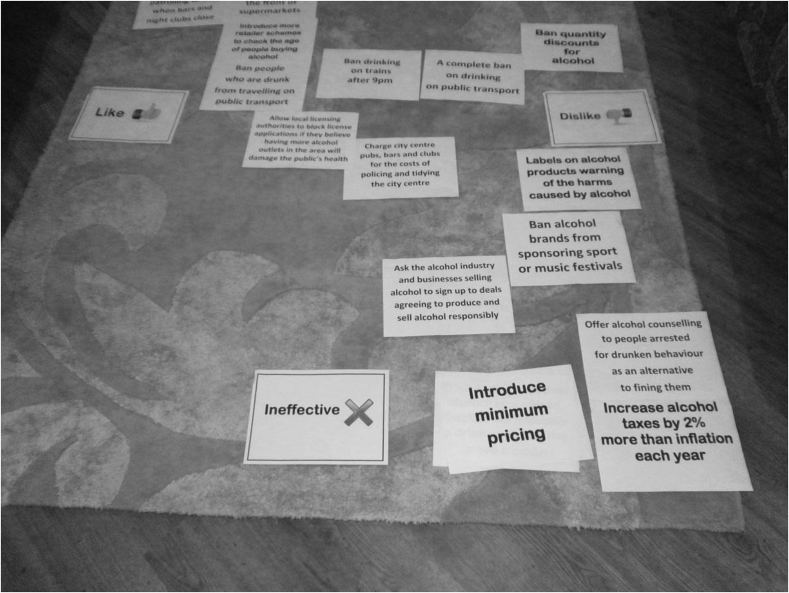


**Table 1:** APISE sample characteristics.^1^

	Group A (N = 1733)		Group B (N = 1727)	
	Unweighted n (%)	Weighted n (%)	Unweighted n (%)	Weighted n (%)
**Gender**				
Male	704 (40.6)	919 (52.6)	688 (39.8)	847 (49.3)
Female	1029 (59.4)	828 (47.4)	1039 (60.2)	872 (50.7)
**Age**				
16-24	94 (5.5)	294 (17.0)	112 (6.5)	324 (18.9)
25-34	215 (12.6)	345 (19.9)	245 (14.3)	374 (21.8)
35-54	877 (51.4)	793 (45.7)	826 (48.2)	704 (41.1)
55-65	519 (30.4)	303 (17.4)	529 (30.9)	311 (18.2)
**Country**				
Scotland	851 (49.1)	159 (9.1)	867 (50.2)	167 (9.7)
England	882 (50.9)	1588 (90.9)	860 (49.8)	1552 (90.3)
**Education**^2^				
None	35 (2.1)	29 (1.7)	49 (2.9)	33 (2.0)
Primary/secondary	296 (17.4)	316 (18.3)	321 (18.9)	303 (17.8)
Secondary advanced/vocational	296 (17.4)	315 (18.3)	279 (16.4)	355 (20.9)
Further education below degree	416 (24.0)	378 (21.9)	425 (25.0)	412 (24.3)
University/post graduate/professional	662 (38.8)	685 (39.8)	628 (36.9)	593 (35.0)
**Annual household income**				
Low (<£20,800)	344 (26.5)	245 (19.2)	310 (23.3)	246 (18.6)
Middle (£20,800–41,599)	466 (35.9)	453 (35.5)	519 (39.0)	495 (37.4)
High (≥£41,600)	487 (37.5)	578 (45.3)	501 (37.7)	582 (44.0)
**Occupation**				
Higher managerial/administrative/professional	686 (50.9)	721 (52.0)	691 (51.1)	695 (52.1)
Intermediate	321 (23.8)	318 (22.9)	293 (21.7)	304 (22.8)
Routine and manual	340 (25.2)	348 (25.1)	368 (27.2)	335 (25.1)
**Children**				
No children	1189 (68.9)	1053 (60.5)	1179 (68.5)	1057 (61.6)
Has children	537 (31.1)	688 (39.5)	543 (31.5)	658 (38.4)
**Drinking**				
Moderate	1083 (62.5)	1039 (59.5)	1054 (61.1)	1015 (59.3)
Hazardous	455 (26.3)	476 (27.2)	457 (26.5)	464 (27.1)
Harmful	194 (11.2)	232 (13.3)	214 (12.4)	233 (13.6)
**People in Scotland/England are generally discouraged or encouraged to drink alcohol**				
Strongly Discouraged	62 (3.6)	57 (3.3)	52 (3.1)	31 (1.8)
Discouraged	191 (11.2)	210 (12.1)	132 (7.6)	99 (5.8)
Neither	603 (35.5)	576 (33.2)	552 (32.0)	536 (31.3)
Encouraged	512 (29.5)	577 (33.2)	597 (35.0)	650 (38.0)
Strongly Encouraged	332 (19.5)	316 (18.2)	371 (21.8)	394 (23.1)
**People in Scotland/England have a very healthy or unhealthy relationship to alcohol**				
Very healthy	34 (2.0)	38 (2.2)	32 (1.9)	36 (2.1)
Healthy	87 (5.1)	80 (4.6)	101 (5.9)	115 (6.7)
Neither	513 (30.0)	562 (32.5)	535 (31.3)	583 (34.1)
Unhealthy	627 (36.7)	690 (39.9)	533 (31.2)	526 (30.8)
Very Unhealthy	447 (26.2)	362 (20.9)	508 (29.7)	450 (26.3)
**The government should do more to tackle the harm done by alcohol**				
Strongly disagree	44 (2.6)	40 (2.3)	47 (2.7)	64 (3.7)
Disagree	201 (11.7)	195 (11.2)	221 (12.9)	221 (12.9)
Neither	227 (13.2)	224 (12.9)	218 (12.7)	207 (12.1)
Agree	668 (38.8)	674 (38.7)	656 (38.2)	671 (39.3)
Strongly Agree	581 (33.8)	607 (34.9)	574 (33.4)	546 (32.0)

^1^ Weighted estimates based on weighted samples combining English and Scottish respondents; Missing categories not shown in table.

^2^ Education full categories - Primary or secondary school/vocational level 1&2/trade apprenticeship (O Grade, Standard Grade, GCSE, GCE O Level); Secondary school advanced/vocational level 3 (A-Levels, AS Levels, Highers); Further Education/training college BELOW degree level (HNC,HND,Diplomes); University/postgraduate degree/professional qualifications.

**Table 2:** Constitution of focus groups.

Group No^a^	Age	Gender	Social Grade^b^	Number attending
E1	16–18	Female	ABC1	6
E2	16–18	Male	C2DE	6
E3	19–24	Female	C2DE	6
E4	19–24	Male	ABC1	5
E5	25–44	Female	C2DE	6
E6	25–44	Male	ABC1	4
E7	45–64	Female	ABC1	6
E8	45–64	Male	C2DE	6
S1	16–18	Female	ABC1	5
S2	16–18	Male	C2DE	6
S3	19–24	Female	C2DE	5
S4	19–24	Male	ABC1	6
S5	25–44	Female	C2DE	4
S6	25–44	Male	ABC1	6
S7	45–64	Female	ABC1	6
S8	45–64	Male	C2DE	6
**Total**				**89**

a ‘E’ denotes English groups; ‘S’ denotes Scottish groups.

b A demographic classification which is standard in the UK and classifies social grades according to occupation was used. ABC1 includes professional/skilled workers and C2DE includes unskilled/manual/unemployed.

**Table 3:** APISE attitudes towards alcohol control policies.

	Strongly Oppose (%)	Oppose (%)	Neither (%)	Support (%)	Strongly support (%)	Mean^a^	n
**Group A**							
Restrictions on numbers of places selling alcohol in your community	9.7	26.0	23.4	24.0	16.9	3.12	1738
Earlier closing times for buying alcohol from off-licenses and supermarkets	13.8	26.2	20.8	21.8	17.4	3.03	1737
An increase in the price of alcohol	18.0	29.4	19.2	21.8	11.6	2.80	1740
Pricing based on alcohol strength so that the stronger a drink is the more it costs	7.7	15.8	13.4	36.6	26.5	3.58	1747
Labels on alcohol products warning of the harms of alcohol	3.0	5.2	10.5	45.8	35.5	4.05	1739
Public information campaigns to raise awareness of harms from alcohol	2.7	3.2	9.6	44.2	40.3	4.16	1741
More treatment services to help dependent drinkers	3.5	5.3	14.4	43.3	33.4	3.98	1733
**Group B**							
An increase in the price of alcohol	19.8	24.0	21.6	24.1	10.4	2.81	1710
Pricing based on alcohol strength so that the stronger a drink is the more it costs	9.4	15.4	18.9	32.8	23.4	3.45	1713
Greater enforcement of laws on under-age sales	1.5	3.2	5.4	29.2	60.7	4.44	1713
Reducing the drink driving limit	19.7	15.0	10.5	23.1	31.7	3.32	1689
Doctors or health professionals asking patients about their drinking habits	1.0	3.7	9.3	45.6	40.3	4.20	1714
A complete ban on drinking on public transport	4.3	11.1	8.7	28.0	47.9	4.04	1715
More police patrolling streets when bars and nightclubs close	2.0	3.3	8.6	39.5	46.6	4.25	1706

a The mean lies on a scale of 1 strongly oppose; 2 oppose; 3 neither support nor oppose; 4 support; 5 strongly support.

**Table 4:** APISE attitudes to alcohol control policies.

Group A	Mean^a^	Group A factor loadings^b^		Group B factor loadings^b^ PCA 1		Group B factor loadings^b^ PCA 2	
		*Restricting availability*	*Provision of health information and treatment services*	*Pricing*	*Reducing anti-social behaviours, harms, and underage sales*	*Pricing*	*Greater law enforcement*
***Restricting availability***	3.12					–	–
Restrictions on numbers of places selling alcohol in your community		**0.763**^c^	0.086	–	–	–	–
Earlier closing times for buying alcohol from off-licenses and supermarkets		**0.775**	0.088	–	–	–	–
An increase in the price of alcohol		**0.777**	0.186	–	–	–	–
Pricing based on alcohol strength so that the stronger a drink is the more it costs		**0.608**	0.306	–	–	–	–
***Provision of health information and treatment services***	4.04			–	–	–	–
Labels on alcohol products warning of the harms of alcohol		0.242	**0.721**	–	–	–	–
Public information campaigns to raise awareness of harms from alcohol		0.220	**0.785**	–	–	–	–
More treatment services to help dependent drinkers		0.023	**0.693**	–	–	–	–
**Chronbach's alpha**		0.748	0.622				
**Group B**							
***Pricing***	3.12						
An increase in the price of alcohol		–	–	**0.815**	−0.010	**0.869**	0.038
Pricing based on alcohol strength so that the stronger a drink is the more it costs		–	–	**0.789**	0.047	**0.850**	0.119
***Reducing anti-social behaviours (ASB), harm, and under age sales***	4.32						
Greater enforcement of laws on under-age sales		–	–	0.102	**0.790**	0.114	**0.833**
Reducing the drink driving limit		–	–	0.304	0.112	–	–
Doctors or health professionals asking patients about their drinking habits		–	–	0.496	0.145	–	–
A complete ban on drinking on public transport		–	–	0.397	0.434	–	–
More police patrolling streets when bars and nightclubs close		–	–	0.051	**0.839**	0.039	**0.853**
**Chronbach's alpha**				0.661	0.454	0.661	0.609

a The mean lies on a scale of 1 strongly oppose; 2 oppose; 3 neither support nor oppose; 4 support; 5 strongly support.

b Factor loadings are from principal component analysis using Varimax rotation. The eigenvalues and percent variance explained by two factors restricting availability and provision of health information and treatment services in Group A were, respectively; 2.88, 41.11%; 1.15, 16.38%. In the first PCA for Group B “PCA 1” for pricing and reducing anti-social behaviours, harms, and underage sales; 2.12, 30.30%; 1.23, 17.54%. In the second PCA for Group B “PCA 2” for pricing and greater law enforcement; 1.73, 43.21%; 1.20, 30.05%.

c Loadings greater than 0.5 marked in bold.

**Table 5:** Unstandardised (B) and standardised (β) coefficients based on multiple linear regression models predicting support for alcohol control policies.

	Group A						Group B					
	Support on Restricting Availability			Support on Health information and treatment services			Support on Pricing			Support on Greater law enforcement		
	B	SE	β	B	SE	β	B	SE	β	B	SE	β
Gender (female)	0.240**	0.041	0.124	0.068*	0.032	0.045	0.190**	0.049	0.086	0.131**	0.036	0.085
Age (ref = 16–24)												
25–34	0.119	0.069	0.049	0.153*	0.055	0.082	0.169*	0.081	0.063	0.495**	0.060	0.264
35–54	0.155*	0.059	0.080	0.162*	0.047	0.107	0.254**	0.071	0.113	0.474**	0.053	0.304
55–65	0.253**	0.071	0.099	0.210**	0.057	0.106	0.264*	0.080	0.092	0.579**	0.060	0.291
Country (England)	0.165*	0.070	0.049	−0.056	0.056	−0.021	0.085	0.081	0.023	−0.044	0.060	−0.017
Education (university+)	−0.099*	0.046	-0.050	−0.011	0.036	−0.007	0.166*	0.055	0.072	−0.185**	0.041	−0.115
Income (high)	−0.143*	0.053	-0.073	−0.205**	0.042	−0.136	−0.102	0.059	−0.046	0.076	0.044	0.049
Income (missing)	−0.122*	0.056	−0.055	−0.100*	0.045	−0.058	−0.006	0.068	−0.002	0.152	0.050	0.083
Occupation (high)	0.007	0.050	0.003	0.137*	0.040	0.090	0.070	0.058	0.031	−0.014	0.043	−0.009
Occupation (missing)	0.127*	0.057	0.052	0.104*	0.046	0.055	0.111	0.067	0.042	−0.116*	0.050	−0.062
Children (yes)	−0.020	0.044	−0.010	0.009	0.035	0.006	−0.065	0.054	−0.029	0.006	0.040	0.004
Drinking (harmful/hazardous)	−0.390**	0.042	-0.197	−0.070*	0.033	−0.046	−0.259**	0.049	−0.116	0.006	0.037	0.004
Encouraged to drink alcohol^a^	0.184**	0.021	0.193	0.092**	0.016	0.124	0.054*	0.027	0.046	0.011	0.020	0.014
Unhealthy relationship^b^	0.080**	0.022	0.076	0.049*	0.018	0.060	0.010	0.026	0.008	0.077**	0.019	0.099
Government responsibility^c^	0.289**	0.020	0.318	0.282**	0.016	0.400	0.394**	0.022	0.403	0.113**	0.016	0.166
Model R^2^	0.301			0.260			0.242			0.132		

**p < 0.01; *p < 0.05 level.

a Would you say that people in Scotland/England are generally discouraged or encouraged to drink alcohol? (1 = strongly discouraged, 5 = strongly encouraged).

b Would you say that people in Scotland/England have a very unhealthy or healthy relationship with relationship with alcohol (1 = very healthy, 5 = very unhealthy).

c The government should do more to tackle the harm done by alcohol (1 = strongly disagree, 5 = strongly agree).
